# Reimagining the role of emotion in healthcare research

**DOI:** 10.1016/j.qrmh.2025.100033

**Published:** 2025-12-11

**Authors:** Rebekah Cole

**Affiliations:** a Department of Military and Emergency Medicine at Uniformed Services University of the Health Sciences in Bethesda, MD, USA; b Department of Health Professions Education at Uniformed Services University of the Health Sciences in Bethesda, MD, USA

**Keywords:** Emotional reflexivity, Qualitative health research, Researcher vulnerability, Emotion in fieldwork, Trauma-informed research, Relational ethics

## Abstract

**Background:**

Qualitative healthcare research often involves emotionally charged topics, such as trauma, illness, loss, moral injury, that profoundly affect researchers as well as participants. Yet the personal and emotional experiences of researchers are frequently excluded from formal training, ethical oversight, and methodological discourse.

**Objective:**

This commentary explores emotional reflexivity as a core methodological, ethical, and pedagogical dimension of qualitative research in health care. It asserts that researcher emotions are vital sources of insight, ethical awareness, and relational depth, particularly in clinical, psychological, and trauma-informed research settings.

**Methods:**

Drawing from feminist standpoint theory, affect theory, and post-qualitative inquiry, this paper synthesizes conceptual literature, cross-disciplinary insights, and a personal fieldwork vignette from qualitative research with Ukrainian military healthcare professionals. Finally, it presents a framework for emotional engagement throughout the research process.

**Results and key insights:**

Emotions shape every stage of qualitative research. When unacknowledged, emotions may contribute to researcher distress or burnout. This commentary highlights the need for emotionally responsive training models, research team practices, and IRB protocols that address participant and researcher vulnerability. It also offers pedagogical strategies and draws parallels to emotionally intensive fields such as counseling, chaplaincy, and medicine.

**Conclusion:**

Emotionally reflexive practice enhances ethical clarity, deepens qualitative rigor, and promotes long-term sustainability in health research careers. As qualitative inquiry continues to shape healthcare policy, education, and practice, researcher emotional engagement must be reimagined as an ethical and methodological asset.

## Introduction: The qualitative researcher as the primary research instrument

In qualitative healthcare research, the researcher serves as the primary instrument for data collection and analysis (Denzin & Lincoln, 2018). This role is especially pronounced in complex clinical contexts, where interviews, observations, and narratives are shaped by the researcher’s relational presence, empathy, and emotional attunement. Unlike quantitative approaches, which often emphasize neutrality and detachment, qualitative inquiry, particularly in the health professions, demands sustained emotional engagement. Researchers may witness suffering, uncertainty, despair, or hope in settings such as hospitals, hospices, conflict zones, or mental health clinics. Such encounters challenge conventional assumptions about objectivity and call for a more reflexive, relational approach to qualitative research methodology (Dickson-Swift et al., 2009, Ellis, 2007).

Despite this potential for emotional impact, training in the health professions and research ethics typically emphasizes participant protections while neglecting the emotional impact of the research process on qualitative researchers themselves. Graduate healthcare professions programs rarely provide guidance on processing grief, vicarious trauma, or moral distress encountered in fieldwork (Dickson-Swift et al., 2008, Rager, 2005). As a result, qualitative healthcare researchers could potentially enter emotionally demanding settings unprepared and unsupported, even as they navigate profoundly human stories.

This commentary begins from the premise that researcher emotional labor is not a distraction, but an asset. To develop this argument, I first trace the historical marginalization of researcher emotions and introduce three theoretical traditions: 1) feminist standpoint theory, 2) affect theory, and 3) post-qualitative inquiry, that all foreground emotion as a source of knowledge. I then present a fieldwork vignette that illustrates how emotional intensity can disrupt and enrich inquiry, followed by a practical framework for understanding emotional engagement across the stages of research. Finally, I consider the pedagogical, institutional, and ethical implications of centering emotional reflexivity in qualitative healthcare research. In structuring the paper in this way, I aim to engage readers from the outset in the importance of emotions and emotional labor, while guiding them step by step through the theoretical and embodied dimensions of the argument.

## Researcher emotion throughout history

To understand why researcher emotions have long been marginalized, it is helpful to trace how scientific traditions historically framed emotion. Throughout the 19th and 20th centuries, emotion was considered incompatible with rigorous research. Researchers were expected to remain neutral observers, detached from their subjects and unaffected by any emotions that emerged during the research process. This ideal of objectivity stemmed from positivist philosophy, which sought to establish the social sciences as legitimate and empirical. In 1853, Comte argued that society could be studied through calculated observation and prediction (Comte, 2009). Durkheim reinforced this vision in 1895 by stating that social facts should be studied objectively (Durkheim, 1982).

In the post–World War II era, this emphasis on detachment intensified with the development of “value neutrality,” which held that researchers must prevent moral or emotional judgments from influencing empirical findings (Weber, 1949). As disciplines strove for scientific legitimacy, emotional restraint became institutionalized. Research ethics committees, textbooks, and peer-reviewed journals reinforced the belief that a researcher’s emotional world should remain separate from the research process (Roulston, 2013). Guided by these philosophies, researchers were trained to protect participants’ emotional well-being, but not to navigate their own emotional responses, which were viewed as weakness or professional failure (Hubbard et al., 2001).

### Theoretical lineage: feminist standpoint theory, affect theory, and post-qualitative inquiry

By the late 20th century, a growing body of scholars began to critique the epistemological violence of a detached approach to research (see Table 1). Feminist epistemologists argued that all knowledge is socially situated and that emotion can itself be a source of insight (Haraway, 1988, Harding, 1991, Jaggar, 1989). Their interventions laid the groundwork for a paradigm shift in which emotional engagement was recognized for its ethical and epistemological value. Three key theoretical traditions shaped this shift: feminist standpoint theory, affect theory, and post-qualitative inquiry. Each positions emotion as central to the production of authentic meaning.

**Table 1:** Summary of theoretical traditions that have redefined the role of emotion in qualitative research.

**Theoretical Tradition**	**Key Contributors**	**Core Concepts**
Feminist Standpoint Theory	Harding (1991);Collins (1990); Jaggar (1989)	Knowledge is socially situated; emotion reveals injustice, power, and marginalization.
Affect Theory	Ahmed (2004); Wetherell (2012); Bondi (2014)	Emotions circulate between bodies and spaces; shape norms, boundaries, and belonging.
Post-Qualitative Inquiry	St. Pierre (2011); Lather (2007); Jackson & Mazzei (2012)	Research is nonlinear and entangled; emotion is central to meaning-making across all phases.

Feminist standpoint theory was among the first to challenge the expectation of researcher objectivity. Harding (1991) and Collins (1990) contended that knowledge is shaped by gender, race, and lived experience, making emotion a valuable lens for recognizing power dynamics and social injustice. Jaggar’s (1989) notion of “outlaw emotions” was particularly influential: Emotions such as anger, fear, and grief can expose social conditions that dominant cultures attempt to obscure (Haraway, 1988).

Affect theory extends the role of emotion beyond individual experience to its collective and systemic impact. Affect theorists argue that emotions do not simply reside within individuals, but circulate between bodies, shaping social norms, group belonging, and political realities (Wetherell, 2012). For instance, Ahmed’s (2004) concept of “emotional economies” describes how feelings such as fear or hope create boundaries between insiders and outsiders. Affect theory encourages attention not only to what participants say, but also to the emotional cues embedded in their silences, gestures, and bodily responses (Bondi, 2014, Wetherell, 2012).

Later in the 21st century, post-qualitative inquiry embraced emotional complexity, uncertainty, and entanglement. St. Pierre (2011) and Lather (2007) rejected the idea that research can be neatly divided into stages of data collection, analysis, and writing. Instead, they advanced an open-ended, cyclical approach in which emotional dimensions are present throughout. Jackson and Mazzei (2012) further argued that emotion is not merely something to reflect on after the fact, but is central to the research process itself.

## Emotional reflexivity in qualitative healthcare research

Applying these theoretical insights to healthcare research, this section illustrates how emotional reflexivity is essential in high-stakes, emotionally charged environments. “Emotional reflexivity” refers to the deliberate practice of attending to one’s affective responses throughout the research process, from project design to fieldwork, analysis, and dissemination (Pillow, 2003). For example, researchers might experience a range of emotions when listening to a terminally ill patient describe their final wishes or when hearing frontline clinicians reflect on burnout or trauma. These emotions shape how questions are posed and how meaning is made (Bondi, 2013, Ellis and Bochner, 2000).

Healthcare researchers often operate within rigid systems that emphasize neutrality and depersonalization. Yet, emotional reflexivity brings forth the researcher’s own values, assumptions, and positionality. This revelation is particularly important in healthcare research that examines suffering, inequality, and/or social determinants of health. During these studies, researcher emotions such as empathy, discomfort, grief, or moral tension could be examined as data that reveal the relational underpinnings of the research encounter (Gilbert, 2001, Josselson, 2013).

Qualitative healthcare researchers are thus called to develop emotional literacy as part of their methodological toolkit. For example, reflective journaling, debriefing with colleagues, and mentorship can support researchers in navigating their emotional responses. Without these supports, emotional labor could remain unprocessed and potentially harmful (Rager, 2005, Wray et al., 2007). By integrating emotional reflexivity into research training, health professions education can better prepare scholars to conduct ethical and emotionally sustainable inquiry. This recognition highlights the need for a structured framework, which I address by outlining four categories of emotional engagement that trace how emotions emerge across the different stages of research.

## Categories of emotional engagement across the research process

To offer practical guidance, I describe four categories of emotional engagement—anticipatory, relational, reflective, and residual—that capture how emotions emerge at different stages of research. This framework provides a clear way to identify, name, and address the wide range of emotions researchers may experience throughout the qualitative research process (see Table 2).

**Table 2:** Emotional engagement framework.

**Category**	**Description**
Anticipatory	Emotions arising before data collection begins (e.g., apprehension, curiosity).
Relational	Emotions experienced during participant interaction (e.g., empathy, discomfort).
Reflective	Emotions that surface during analysis and writing (e.g., grief, pride).
Residual	Emotions that linger after the study concludes (e.g., moral conflict, resentment, burnout).

### Anticipatory emotion

Anticipatory emotion could potentially encompass the emotions a researcher brings to the study even before entering the field. These emotions may include excitement, curiosity, nervousness, apprehension, or even dread. These emotions might stem from the researcher’s personal identity, location, past lived experiences, and relationship to the phenomenon under study (Finlay, 2002a, Peshkin, 1988). For example, a researcher studying marital conflict who has recently experienced a divorce might carry both a sense of solidarity and heightened vulnerability into the research space.

Ultimately, anticipatory emotions might shape how we approach participants and design a study. Researchers could feel tension about whether they are ready to engage a sensitive topic or whether their emotional proximity could cloud their judgment (Watts, 2008). Yet, such concerns also carry the potential for ethical insight. Recognizing anticipatory emotions might spark pre-fieldwork reflexivity, allowing researchers to examine and process what they are bringing into the research process (Bott, 2010).

### Relational emotion

Relational emotion is most felt during interactions with participants during interviews, focus groups, or participant observations. These feelings might include trust, empathy, or discomfort (Oakley, 1981, Rager, 2005). For example, a researcher might feel emotionally overwhelmed during an interview with a trauma survivor. Relational emotions can raise important ethical and epistemological concerns, including how to stay emotionally present without over-identifying with participants or how to respond to emotional disclosures with care while preserving appropriate boundaries. They can also reveal power imbalances, hidden assumptions, and unspoken tensions that require continued self-reflection (Campbell, 2002, Etherington, 2007).

### Reflective emotion

Reflective emotions arise after data collection concludes, potentially during transcription, data analysis, writing, and/or dissemination. These emotions can surface after researchers have time to fully absorb what they have seen or heard (Dickson-Swift et al., 2008). For example, researchers may find themselves becoming emotional while transcribing a particular interview as they listen to a particularly painful story over and over again. Reflective emotions may also include positive emotions such as joy, reverence, pride, or a sense of purpose, especially when analysis uncovers stories of resilience, transformation, or hope (Ellis, 2007). These emotions also deserve attention as to what matters to the researcher, and how this mattering may be impacting the research process.

### Residual emotion

Residual emotions linger after the study is complete. These emotions can take many forms such as grief, unresolved moral tension, burnout, pride, attachment, or even longing (Coffey, 1999). They often reflect the enduring psychological and ethical imprint the research has left on the researcher’s sense of self and worldview. Residual emotions are often less visible to outsiders, potentially surfacing months or years after the research study has concluded.

Residual emotion can be an underrecognized threat in qualitative inquiry. It poses risks to researchers’ mental health and ethical clarity when unacknowledged or unsupported. Creating opportunities for qualitative researchers to process residual emotion through mentorship, peer debriefing, or therapy could foster a more humane, sustainable, and relationally attuned qualitative research culture (Raheim et al., 2016, Wray et al., 2007). While this framework offers structure, actual fieldwork rarely follows neat stages. The next section addresses the unpredictable and disruptive realities of emotional engagement.

## The emotional realities of fieldwork

While emotional engagement in qualitative research can be conceptualized in stages—anticipatory, relational, reflective, and residual—these categories are not necessarily set in stone or predictable. Once in the field, researchers may encounter emotional experiences that are unpredictable or shaped by unique contexts. For example, emotional responses may intensify or appear suddenly in response to an interaction with a participant (Bondi, 2014, Ellis and Bochner, 2000).

### Unanticipated emotional triggers

Qualitative researchers might expect certain topics to be difficult; however, moments that trigger emotional responses can be unanticipated. For example, a participant's tone of voice or a smell in a back room might strike with unexpected emotional intensity (Coffey, 1999, Pezalla et al., 2012). Rather than serving as distractions, these emotional disruptions, when met with thoughtful reflection, can yield valuable insights for both the participant and the researcher (Bondi, 2014, Ellis and Bochner, 2000).

### Environmental emotion

Fieldwork is not only shaped by the researcher-participant interactions, but also by the setting. The surrounding environment such as hospitals, military bases, or disaster sites can influence how researchers experience and understand emotion. Sights, sounds, smells, or even the weather can impact emotional responses. These details can be considered a part of the research process that influences the researcher’s meaning making and approach to answering the research question (Desjarlais, 1997, Pink, 2009). Researchers might also feel emotional responses in their bodies. For example, shoulder tension, sudden fatigue, or a shortness of breath may signal discomfort or resonance. Paying close attention to these somatic responses and processing them with a research team can potentially enrich data analysis (MacLure, 2013a, St. Pierre, 1997).

### Temporal dissonance and emotional echoes

Finally, the emotional impact of fieldwork might not be immediately noticeable. Some researchers have described an unexpected emotional “aftershock” both in the short and long term after research is complete (Rager, 2005). This time delay can impact how researchers process and make sense of their experience as a seemingly ordinary interaction might later feel unsettling. Or, a moment that felt flat or awkward might later reveal emotional depth. As a result, emotional processing and reflexivity might need to continue throughout the research process, and, potentially, after the study has ended.

## Emotions as potentially disruptive forces in qualitative methodology

While emotion is often discussed as a positive force in qualitative research, it is also important to discuss how emotional responses can sometimes disrupt the research process. For example, when a participant discloses something deeply painful or triggering, the researcher might switch to a role of providing emotional care (Campbell, 2002, Dickson-Swift et al., 2009). In other cases, researchers might find themselves unable to continue with the research project due to their emotional exhaustion, vicarious trauma, or ethical or moral discomfort (Rager, 2005).

These disruptions are not methodological failures; rather, they reflect the emotional complexity of qualitative research. For example, Dickson-Swift et al. (2008) describe researchers interviewing people about terminal illness or traumatic loss who experienced distress that led to pauses in data collection, difficulty maintaining objectivity, or even withdrawal from the study. Such emotional detours are often left out of methodological reports. This silence can limit support for researchers and reinforce the false expectation that qualitative studies should unfold without flexibility (Coles & Mudaly, 2010). Normalizing and openly discussing these experiences can enrich methodological literature by offering a more authentic representation of qualitative work (Ellis & Bochner, 2000).

Keeping in mind the potential of emotions to impact the researcher and the research process, the following vignette is a personal account from a research encounter that reshaped not only how I understood my participants’ stories, but how I understood my role, my limits, and my own emotional threshold as a qualitative researcher.

## Personal vignette

The following account from my fieldwork with Ukrainian military healthcare professionals illustrates how unanticipated emotional intensity reshaped both the interview process and my understanding of qualitative inquiry. Last November, I traveled to Poland to conduct a qualitative study interviewing Ukrainian military healthcare professionals who were engaged in the Ukrainian-Russian war. So far, their experiences had not been studied, but only reported in the media. This was a new frontier that I was eager to explore. I know their stories would be intense, but I was not prepared for the level of emotional intensity that I felt as I sat across from each of them and they recounted the horrors of the battlefield. They spoke of the pain of watching their comrades die. They described their own deaths as inevitable, accepting their fate. Despite their own suffering, they described efforts to shield their loved ones from the horror of their experiences. The stories I heard resembled trauma narratives encountered in clinical mental health, palliative care, and humanitarian medicine.

As I listened to each of these interviews, I tried to remain a professional, stoic, and an active listener. However, during the eighth interview, as my participant’s story began to develop, a dam burst. I began to weep in the middle of the interview, tears pouring down my face. I cried for my participant’s pain and suffering, for the people in her country, for her family, and for the fact that there was no end in sight for the horrors of war that they were experiencing. I mourned her death, even though she sat very much alive right in front of me.

At that moment, I took a slow, steady breath to compose myself—no longer just a qualitative researcher, but fully human, deeply connected to the weight of my participant’s story. And in that instant, I believe she felt that shift too, because she reached over and held my hand. We sat together in silence for a few moments before she continued with her story. From that moment on, my probing questions felt less automatic—no longer just, “Tell me more about that,” but emerging from a deeper, more vulnerable place. At one point, I took a deep breath and asked, “How are you so brave?”

When the interview ended, I was emotionally, mentally, and physically exhausted. I sat still in the empty room and felt an immediate sense of doubt and regret. Had I failed as a qualitative researcher? Had I disrespected the space I was trained to hold so sacredly for my participant? At that moment, I doubted my work as a researcher and the very nature of qualitative inquiry. Yet, as I reflected, I began to question whether my emotional response had, in fact, contributed to a richer, more profound research encounter. This vignette exemplifies how emotion can disrupt research conventions, but also deepen relational connection, raising broader questions about how emotional responsiveness can be harnessed methodologically.

## Harnessing emotionalism in the research process

As in my vignette, qualitative researchers might try to set aside their emotional reactions to approach participants’ narratives with greater objectivity and to minimize bias or the projection of personal perspectives onto the data. However, when the data involve deeply personal accounts of trauma, loss, injustice, or resilience, emotional suppression may not be the best approach. Leaning into and exploring one’s emotions can foster a deeper connection with, and understanding of, participants’ experiences. Holmes (2020) argues that ignoring emotional impact risks losing the richness of qualitative data. In emotionally charged encounters—such as my work with Ukrainian healthcare professionals—efforts to suppress or contain emotion can undermine the authenticity of the researcher–participant relationship, potentially eroding relational trust (Dickson-Swift et al., 2009, Johnson and Clarke, 2003).

In contrast, allowing space for authentic emotional expression, when paired with reflexivity and ethical consideration, can make the interaction more meaningful and productive (Etherington, 2007). Emotional openness can also validate participants’ experiences, signaling that their stories are not only heard, but also felt and appreciated. In this way, emotional responsiveness can strengthen the depth of the researcher–participant relationship and enhance the overall quality of the data (Gilbert, 2001, Hubbard et al., 2001).

However, embracing emotional expression must be accompanied by reflection and bracketing of one’s preconceived notions and beliefs that may be intermixed in the emotional response to avoid the risk of centering the focus of the interview or focus group on the researcher’s emotions rather than the participant’s narrative. Pillow (2003) cautions against the dangers of such displays of emotion that shift the focus of the research encounter around the researcher, rather than on the participants’ experience. Ulimtately, this distraction can disrupt the power dynamics of the interview and cause ethical complications.

A balanced approach is essential, neither suppressing emotions nor allowing them to run unchecked. Reflexivity supports this balance by helping researchers examine their emotional responses in context, consider how those responses affect their relationships with participants, and evaluate whether emotion enriches or hinders the inquiry (Finlay, 2002a, Tracy, 2010). Research training and development can help students learn how to recognize and manage their emotions while maintaining appropriate ethical boundaries. Because these skills are complex, they develop gradually and require ongoing practice, mentorship, and supervision throughout a researcher’s career.

## The role of emotion in qualitative data analysis and manuscript writing

When considering the importance of reflexivity, it is crucial to recognize that qualitative researchers might experience powerful emotions beyond the data collection stage. For example, transcribing interview or focus group data may evoke grief, anger, or deep empathy (Dickson-Swift et al., 2009). During data analysis, these emotional reactions can subtly shape coding decisions and recognition of themes (Saldaña, 2015). Direct quotes that provoke a strong emotional response may feel more salient or significant, potentially leading researchers to overemphasize certain themes (Pezalla et al., 2012). Conversely, data that triggers discomfort or guilt might be avoided (Pillow, 2003).

To mitigate these influences, researchers are encouraged to debrief with their team about their emotional reactions and how those reactions might be affecting the study’s findings, either positively or negatively (Watts, 2008). Involving multiple team members from diverse backgrounds can also provide critical perspectives and help identify salient themes and patterns with greater balance (Finlay 2002a).

Emotions can similarly shape the writing process, influencing tone, voice, and decisions about what data to include or omit (Ellis & Bochner, 2000). Emotional engagement during writing raises ethical considerations: Researchers must convey participants’ meanings while ensuring their own emotions do not overshadow participants’ voices. Member checking offers one potential solution, helping to confirm that emotionally resonant narratives remain ethically sound and accurately represent participants’ experiences (Lincoln & Guba, 1985).

## Training and pedagogical gaps

Although qualitative methods are increasingly embedded in medical education, nursing, public health, and clinical psychology programs, the emotional demands of qualitative research are seldom addressed in formal training. Graduate students are typically taught to ensure participant safety, adhere to ethical review standards, and maintain methodological rigor. Yet, few are prepared for the emotional intensity of conducting research on health-related suffering, (i.e., topics such as trauma, illness, death, and systemic injustice) (Dickson-Swift et al., 2009, Rager, 2005).

This gap in training is particularly concerning, given parallels between emotionally intensive research encounters and clinical care experiences. Like healthcare professionals, qualitative researchers often witness human vulnerability in deeply personal and relational ways. However, while healthcare professionals are typically trained in emotional regulation, boundary setting, and reflective supervision, researchers are rarely afforded the same scaffolding. Consequently, when researchers experience vicarious trauma, moral distress, or emotional burnout, they might feel ashamed, isolated, or unprofessional, potentially misinterpreting these natural responses as methodological failures rather than as indicators of ethical attunement (Coles and Mudaly, 2010, Guillemin and Gillam, 2004).

Ingraining emotional reflexivity within qualitative methodology curricula for health professions can help address this gap. This integration includes not only theoretical discussion of emotion in research, but also structured tools for emotional processing and skill development. As shown in Table 3, pedagogical strategies could include reflective journaling, peer debriefing, and ethical case studies. Simulation-based interviews could likewise be adapted to help students practice navigating affectively charged research encounters (Hutchinson et al., 2023). Moreover, coaching and mentorship might be considered to be an integral part of this training. In these relationships, faculty can model ethical responses to emotional complexity by sharing their own experiences in navigating emotionally difficult fieldwork, clinical interviews, and/or data analysis.

**Table 3:** Pedagogical strategies for developing emotional reflexivity in qualitative research.

**Training Strategy**	**Purpose**	**Reference**
Reflective journaling	Supports data analysis and provides a space to process thoughts and feelings.	Watt (2007)
Peer debriefing groups	Offers structured, supportive spaces for researchers to share and normalize emotional challenges.	Rager (2005)
Ethical case studies	Explores researcher vulnerability, moral distress, and emotional ambiguity in ethical contexts.	Guillemin and Heggen (2009)
Simulated interviews and focus groups	Builds emotional intelligence through role-play and guided reflection on emotional dynamics.	Hutchinson et al. (2023)
Training on emotional impact	Prepares researchers to manage vicarious trauma, moral injury, and compassion fatigue.	Coles and Mudaly (2010); Bressi and Vaden (2017)

Emotional reflexivity can also be integrated throughout the entire research methodology curriculum. For instance, coursework on epistemology and methodology could critically engage with feminist, postcolonial, and affect theory traditions that highlight the role of emotion in knowledge formation (Ahmed, 2004, Pillow, 2003). Discussions of methodological rigor could include emotional and relational aspects. Assignments could invite students to explore their own emotional responses to interviews, stories, or case studies and then engage in ongoing reflection about how to best receive and process these emotions.

Mentors might likewise model healthy and ethically sound approaches to navigating emotional complexity. Faculty who openly share examples of their emotional experiences and decision-making processes during fieldwork normalize this reaction and demonstrate that reflection is an important part of the research process (Ellis et al., 2011, Finlay, 2002a). Mentors can encourage emotional check-ins, offer validation, and provide resources to help students develop their own strategies for emotional self-care and resilience.

Ultimately, addressing training and pedagogical gaps is not only about researcher well-being, but about protecting the integrity of the research process. Emotions shape what questions we ask, how we hear and interpret responses, and the kinds of knowledge we produce (Holliday, 2007, Josselson, 2007). By teaching researchers to develop emotional reflexivity and resilience, we better equip them to conduct ethical and methodologically sound research.

## Emotional care for participants and researchers in qualitative research

When working with emotionally complex topics or vulnerable populations, qualitative researchers might consider how the structure and pace of the study can promote emotional safety and ethical responsiveness for both the researcher and the participant. Emotionally responsive design begins with attending to the interview environment. Past research has noted that the physical space in which data is collected can profoundly shape emotional comfort and relational dynamics (Bondi, 2014, Ellis, 2007). Small adjustments, such intentionally thinking about the location of interviews or offering opportunity for participants to take breaks, can foster a more emotionally attuned participant-researcher relationship (Campbell, 2002, Dickson-Swift et al., 2008).

Moreover, research protocols can include built-in protocols for emotional care for both the researcher and participants. For participants, these practices could include allotting time to reflect or decompress afterward, or scheduling follow-up check-ins to support ongoing consent and emotional processing (Hutchinson et al., 2023, Rager, 2005). For researchers, emotionally sustainable practices might include structured debriefing with colleagues or research team, reflective journaling, or engaging in supervision or counseling during emotionally intense fieldwork (Coles and Mudaly, 2010, Etherington, 2007). These practices support well-being and can also enhance methodological rigor by fostering ongoing reflexivity and ethical clarity.

Researchers might also need flexibility when emotional dynamics demand it. For example, researchers may need to postpone an interview when a participant expresses distress, modify the study protocol to protect a participant’s emotional boundaries, or redefine the scope of the research when it becomes emotionally unsustainable (Wray et al., 2007). These adjustments can promote responsive, relational, and ethically engaged research practice. To design emotionally sustainable studies, researchers might think not only about their methods and protocols, but also about the training, support systems, and disciplinary cultures that shape emotional engagement.

## Cross-disciplinary parallels

Counseling, chaplaincy, medicine, and social work provide models for cultivating emotional reflexivity that can inform qualitative training. These fields have long recognized that human encounters are emotionally charged, ethically complex, and potentially psychologically taxing. As a result, their training programs provide rich models for cultivating emotional literacy, reflective practice, and ethical resilience that qualitative researchers can draw upon to better navigate their own emotional landscapes.

First, in clinical social work and psychology, emotional boundaries, supervision, and countertransference are explicit components of training. Practitioners learn to recognize how their emotional responses influence client care, and they receive regular supervision to process difficult encounters. These reflective practices mirror the kinds of emotional reflexivity qualitative researchers need when working with participants who share stories of trauma, chronic illness, or end-of-life care (Geller and Greenberg, 2012, Gelso and Hayes, 2007). Structured support is viewed not as remediation, but as essential to ethical, sustainable practice.

Similarly, counselor education programs emphasize empathy, emotional presence, and the therapeutic alliance with clients. Through live supervision, reflective journaling, and in-depth processing of client interactions, counseling students learn to manage emotional engagement (Corey, 2016, Rogers, 1957). These principles are directly applicable to qualitative health research, where deep listening and empathic attunement are central to ethical inquiry.

Chaplains offer another model for consideration. They are prepared to effectively address suffering in medical and crisis settings such as hospital rooms, military combat zones, and disaster sites. In order to manage these intense emotional experiences, Chaplains emphasize narrative containment, spiritual presence, and reflective peer dialogue (Doehring, 2015, Fitchett and Nolan, 2015). These are critical tools for researchers engaging in qualitative interviews on topics such as moral injury, grief, or spiritual distress in healthcare.

In the healthcare professions, emotionally intensive encounters are acknowledged as part of professional development. For example, palliative care training includes communication strategies for managing difficult conversations, self-care modules to mitigate compassion fatigue, and reflective rounds to process emotional cases. While qualitative researchers may not deliver clinical care, they often listen to similar narratives of suffering, resilience, uncertainty, or unfixable problems, and they, too, may need space to reflect and recalibrate.

Finally, medical anthropology and global health research have long recognized the emotional toll of immersion in communities experiencing structural violence, health inequity, or disaster. Anthropologists are trained to anticipate emotional strain and to actively reflect on the positional and affective dynamics of fieldwork (Davies & Spencer, 2010).

Across these fields, there is a shared recognition that emotional engagement is not only expected, but must be supported. Supervision, emotional debriefing, and reflective practices are built into professional cultures to safeguard practitioners and ensure ethical responsibility. Integrating these cross-disciplinary models into qualitative healthcare research training would offer much-needed tools for researchers without the institutional support typically available in clinical or counseling contexts.

## The role of emotion in team-based qualitative research

Qualitative healthcare research teams often draw members from medicine, nursing, psychology, public health, and the social sciences. Each brings distinct epistemologies, positionalities, and emotional orientations to the data. These affective responses influence how data are analyzed and which themes are highlighted or overlooked (Boylorn and Orbe, 2014, Ellingson, 2009). Differences in emotionally charged interpretations can generate tension. A narrative about clinician burnout, for instance, might prompt protectiveness or defensiveness in one team member and a call for structural reform in another. Without deliberate opportunities for emotional reflection, such differences can create friction. Teams can mitigate this friction by leveraging scheduled debriefings, reflective check-ins during coding meetings, and/or explicit agreements on how to handle distressing content.

## Institutional and ethical implications

Institutional Review Boards (IRBs) play a critical role in safeguarding the rights and welfare of research participants, particularly in health-related studies. However, in qualitative healthcare research, especially those involving trauma, terminal illness, grief, or structural inequities, emotional risk is not limited to participants. Researchers are exposed to significant affective burdens, particularly when they conduct in-depth interviews, analyze distressing narratives, or engage in long-term relational fieldwork. Despite these potential threats, most IRBs focus almost exclusively on participant harm and rarely require researchers to assess or mitigate emotional risks to themselves (Dickson-Swift et al., 2008, Haverkamp, 2005).

This oversight reflects a legacy of positivist assumptions that frame the researcher as a neutral observer, unaffected by the stories they encounter. In clinical and healthcare research contexts, such assumptions are especially problematic. Qualitative healthcare researchers often engage directly with patients, caregivers, or healthcare professionals recounting experiences of suffering, loss, and moral injury. These narratives can leave lasting psychological imprints on the researcher, particularly when the researcher shares aspects of identity or experience with their participants (Coles and Mudaly, 2010, Raheim et al., 2016). Without formal acknowledgement of these risks, researchers may experience moral distress, emotional exhaustion, or even burnout, with little institutional support in place.

To address this ethical gap, IRBs, particularly those housed within academic medical centers and schools of public health, should expand their protocols to include researcher well-being as a legitimate dimension of ethical review. Specifically, IRBs could require that investigators:

• Acknowledge potential emotional risk to the research team in studies involving sensitive topics
• Articulate a plan for managing researcher distress, including peer debriefing, supervision, or access to counseling support
• Provide evidence of researcher preparation, particularly for trainees or early career investigators working in high-stakes emotional contexts
• Commit to post-study emotional debriefs or structured reflection sessions as part of study closure

## Complexities of emotionalism within the participant-researcher relationship

Qualitative researchers must manage a balance between emotional openness and professional composure. On one hand, emotional presence fosters empathy, connection, and relational depth, which are essential qualities for building trust with the participant and generating rich, meaningful data (Dickson-Swift et al., 2008, Ellis, 2007). Conversely, researchers are expected to maintain a degree of emotional detachment to ensure analytic clarity, uphold institutional norms of professionalism, and avoid over-identification with participants (Hubbard et al., 2001). Another paradox exists in asking participants to be vulnerable and to share their inner thoughts and feelings, while at the same time, researchers might feel hesitant to reciprocate or process emotional responses openly for fear of compromising credibility or taking attention away from the participant (Bondi, 2013, Gilbert, 2001). This inner conflict may result in guilt, shame, or self-censorship, particularly when researchers feel they have either overstepped or failed to adequately process the emotional depth of the encounter with the participant (Rager, 2005).

Ellis (2007) described this dilemma in her work on autoethnography, exploring whether a researcher's emotional expression, such as crying during an interview, creates space for mutual recognition or risks re-centering the encounter around the researcher. These questions are further complicated by power differentials between researcher and participant, especially in contexts involving trauma or marginalization (Campbell, 2002). In such cases, Ellis posits that emotional reciprocity can build solidarity, but may also create expectations for support or emotional caregiving that extend outside the boundaries of the research relationship. Further complicating this terrain are the institutional and disciplinary norms that have traditionally privileged stoicism and emotional restraint (Hochschild, 1983, Pillow, 2003). Within these norms, researchers might be discouraged from acknowledging the emotional or mental costs of their work (Dickson-Swift et al., 2009).

These complexities require continuous reflexivity throughout the research process. Engaging with these tensions also means recognizing that emotional responses can be shaped by social position, societal expectations, and institutional power (Katz, 1994, Sangtin Writers Collective and Nagar, 2006). For instance, qualitative researchers from historically marginalized communities might experience unique pressures around navigating stereotypes that label them as too emotional, too close to their topic, or not objective enough (Johnson et al., 2020).

## The researcher’s emotional afterlife: Emotion and career longevity

The emotional labor of qualitative research does not end when a study concludes. Residual emotions could potentially accumulate across careers, shaping researcher identity and sustainability. Repeated immersion in painful topics such as trauma, injustice, grief, or systemic violence can lead to vicarious trauma, emotional exhaustion, and eventual burnout (Dickson-Swift et al., 2009, Rager, 2005). Unlike clinical professions where supervision is a norm, qualitative researchers often navigate the emotional toll of their work on their own, without any institutional intervention or support (Figley, 1995, Hochschild, 1983).

Residual emotions from one study can carry into subsequent projects, influencing how researchers engage with new participants, design interview protocols, or interpret data. This emotional carryover can provide ethical and epistemological insight, as past experiences enhance sensitivity and attunement (Etherington, 2007, Finlay, 2002a). However, without deliberate reflection and integration, lingering emotions may create ethical ambiguities, blur analytic clarity, foster over-identification with participants, and/or generate resistance to challenging data (Campbell, 2002, Kisfalvi and Pitcher, 2003).

Given these risks, qualitative researchers sometimes need to develop intentional practices for revisiting and re-evaluating the emotional impact of their work. Lifelong emotional reflexivity could include periodic reflective writing, revisiting old fieldnotes, or processing emotional memory with trusted coaches, mentors, or peers (Pillow, 2003, Ellis, 2007). Some researchers engage in creative forms of representation such as autoethnography, performance, or arts-based methods to process and understand residual emotions (Ellis and Bochner, 2000, Leavy, 2015). Others might capitalize on their accumulated emotional knowledge to mentor students with greater empathy and realism about the affective challenges of the field. Ultimately, career longevity in qualitative research will depend not only on intellectual rigor or institutional support, but also on the ability of researchers to recognize and process their emotions throughout their career.

## Implications for healthcare and medical research training

Few studies have examined how researchers engage in emotional reflexivity across different qualitative methodologies. Comparative studies could investigate how emotional processing varies across qualitative traditions (e.g., phenomenology vs. narrative inquiry), or how emotional responses evolve at different points in the research process. Mixed-methods or autoethnographic designs can be especially helpful for tracing these emotional trajectories and examining their impact on research outcomes.

In addition, there is currently a lack of pedagogical training in emotional literacy and reflexivity within qualitative methodology programs (Dickson-Swift et al., 2009, Finlay, 2002a, Pillow, 2003). However, little is known about which types of instruction are most effective. Future studies could evaluate formal curricular innovations such as reflective writing, simulated interviews/focus groups, or peer debriefing groups to assess their impact on emotional preparedness, ethical sensitivity, and researcher well-being. Research could also explore how faculty mentorship shapes students' emotional resilience and professional identity formation (Etherington, 2007, Rager, 2005).

Qualitative healthcare research, especially on sensitive or traumatic topics, can take an emotional toll on researchers (Campbell, 2002, Dickson-Swift et al., 2008, Rager, 2005). However, few studies document the longer-term mental health impact of qualitative fieldwork. Future research might explore how researchers cope with emotional exhaustion, vicarious trauma, moral distress, and how institutions can develop sustainable structures of care and support for research communities.

Future research is also warranted regarding how emotional engagement varies among researchers. For example, researchers from historically marginalized backgrounds may carry layered emotional burdens when conducting research in communities to which they are personally connected. Research is therefore needed examining how race, gender, sexuality, class, and other identities handle emotional experience within the research process and how institutional or cultural norms might impact these experiences (Collins, 1990, Haraway, 1988, Jaggar, 1989, Raheim et al., 2016).

Because Institutional Review Boards (IRBs) rarely assess emotional risk to researchers, further research could investigate how ethical oversight bodies might be restructured to include sections on researcher well-being (Kisfalvi and Pitcher, 2003, Wray et al., 2007). Studies could explore how IRB protocols could incorporate assessments of emotional risk, support systems, and follow-up care. This additional oversight could advance both methodological rigor and ethical responsibility.

Finally, researchers might continue exploring how emotion might be treated as data in its own right, exploring how both positive and negative emotions shape data analysis and interpretation and the writing process (Ahmed, 2004, Jackson and Mazzei, 2012, MacLure, 2013b, St. Pierre, 2011). This insight could deepen our understanding of the emotional dimensions of meaning-making and offer new models for emotionally aware data analysis.

## Conclusion: Reimagining emotion as a methodological asset in healthcare research

This commentary began from the premise that researcher emotional labor is not a distraction, but a methodological and ethical asset. To develop this argument, I traced the history of researcher emotion, introduced key theoretical frameworks, presented a fieldwork vignette, proposed a framework of emotional engagement, and explored pedagogical and institutional implications. Taken together, these steps demonstrate that emotion is not a liability to be managed or suppressed, but a generative force that can enhance reflexivity, deepen ethical clarity, and sustain qualitative health research.

Emotion is intrinsic to qualitative inquiry in medicine and healthcare, particularly in studies addressing trauma, illness, grief, caregiving, or moral injury. Recognizing and reflexively engaging with these emotions is a potential source of ethical sensitivity, relational integrity, and analytical depth. Emotional reflexivity might therefore be integrated into qualitative training programs, research team practices, and institutional ethics protocols. Doing so would strengthen methodological rigor and protect researcher well-being and foster sustainable careers in emotionally demanding fields.

To advance qualitative inquiry in healthcare, educators, mentors, and institutions should consider emotion as central, not peripheral, to knowledge creation. Building structures of care, reflection, and support for emotionally engaged researchers will lead to more humane, ethically sound, and methodologically robust scholarship. As healthcare systems increasingly rely on qualitative evidence to shape practice and policy, emotionally attuned researchers will be better equipped to understand and convey the human experience.

## Ethical approval

N/A

## Disclaimer statement

The opinions and assertions expressed herein are those of the author and do not reflect the official policy or position of the Uniformed Services University of the Health Sciences or the Department of War.

## CRediT authorship contribution statement

**Rebekah Cole:** Writing – review & editing, Writing – original draft, Conceptualization.

## Declaration of Generative AI and AI-assisted technologies in the writing process

Portions of this manuscript were supported by the use of ChatGPT (OpenAI) for copy editing, organizing content, formatting, and refining text. All content was reviewed, verified, and substantively revised by the author to ensure accuracy, originality, and alignment with scholarly and ethical standards.

## Declaration of competing interests

The author declares no known competing financial interests or personal relationships that could have appeared to influence the work reported in this paper.
